# Temperature Measurement of Fluid Flows by Using a Focusing Schlieren Method

**DOI:** 10.3390/s19010012

**Published:** 2018-12-20

**Authors:** A. Martínez-González, D. Moreno-Hernández, J. A. Guerrero-Viramontes, M. León-Rodríguez, J. C. I. Zamarripa-Ramírez, C. Carrillo-Delgado

**Affiliations:** 1 Depto. de Ingeniería Robótica, Universidad Politécnica del Bicentenario, Carr. Silao-Romita Km 2, San Juan de los Duran, C.P. 36283 Silao, Guanajuato, Mexico; ccarrillop@upbicentenario.edu.mx; 2 TecNM/Instituto Tecnológico de Aguascalientes, Adolfo López Mateos 1801, 20256 Aguascalientes, Mexico; jaguerrero@mail.ita.mx; 3 Centro de Investigaciones en Óptica A.C., Loma del Bosque 115, Lomas del Campestre, CP 37150 León, Guanajuato, Mexico; jzamarripa@cio.mx; 4 Universidad Politécnica de Guanajuato, Av. Universidad sur 1001, C.P. 38496 Cortázar, Guanajuato, Mexico; y_migue@hotmail.com

**Keywords:** focusing schlieren, temperature, measurements

## Abstract

A method for measuring planar temperature fields of fluid flows is proposed. The focusing schlieren technique together with a calibration procedure to fulfill such a purpose is used. The focusing schlieren technique uses an off-axis circular illumination to reduce the depth of focus of the optical system. The calibration procedure is based on the relation of the intensity level of each pixel of a focused schlieren image to the corresponding cutoff grid position measured at the exit focal plane of the schlieren lens. The method is applied to measure planar temperature fields of the hot air issuing from a 10 mm diameter nozzle of a commercial Hot Air Gun Soldering Station Welding. Our tests are carried out at different temperature values and different planes along the radial position of the nozzle of the hot air. The experimental values of temperature measurements are in agree with those measured using a thermocouple.

## 1. Introduction

The interest of temperature measurement is important in a wide area of science and the optical full field methods are preferred to fulfill this important task. The optical techniques preferred to measure temperature in transparent media are Planar Laser Induced-Fluorescence (PLIF), Planar Laser-Induced Phosphorescence (PLIP), interferometry, holography, laser speckle photography and the schlieren technique [1]. 

Schlieren technique is one of the most successful optical method used to visualize fluid flows. This technique has remarkable advantages like easy to implement, uses conventional light sources, is low cost and is very sensitive [2,3]. This method has been improved to face different types of problems in science and engineering [4,5,6,7,8,9,10,11,12,13,14,15,16,17,18,19,20,21,22,23]. Even though initially it was used as a visualization method, it has been used to quantify temperature data in fluid flows [8,9,10,11,12,13,14,15,16]. Nowadays different variant of the optical system has been implemented such as color schlieren [10,11,12], background oriented schlieren [13,14] and calibration schlieren [15,16]. This method has allowed to measure simultaneously temperature and velocity in fluid flows [19,20,21,22]. 

A drawback of schlieren technique is that provides information integrated along the optical path. Therefore, for a three-dimensional temperature field, the values are not so accurate since a two-dimensional analysis technique is applied, this is because the schlieren method averages the values of the refractive index along the thickness of the object under analysis making local temperature measurement difficult. However, is not the case for a focusing imaging technique in which it is possible to visualize only the refractive index disturbances in a limited volume along the optical path. Thereby, focusing schlieren systems is useful to explore a three-dimensional refractive field by incrementally visualizing individual planes along and perpendicular to the optical path. In the same context, Planar Laser Induced-Fluorescence (PLIF) and Planar Laser-Induced Phosphorescence (PLIP) are attractive techniques to measure velocity/temperature [24,25,26,27,28]. PLIF and PLIP provide the ability resolving spatially and temporarily unsteady fluid flow features. These techniques requires a medium to visualize called tracers that are excited with a light source at certain wavelength. The excited tracers (atoms or molecules) emit light spontaneously usually in at a larger wavelength which intensity is registered on a digital camera. There are different implementations of these techniques [24]. However, to calculate physical parameters from the measured signal, the optical set-up and laser tuning become rather complicated and difficult to apply in flows in real conditions. These techniques in general become expensive [24,28]. However, the techniques offer precision values around ~3% [28].

The focusing schlieren methods allow to achieve a narrow depth of focus useful to obtain planar temperature measurements in three-dimensional fluid flows. The focusing image techniques for studying fluid flows have been previously explored for several authors [5,23,29,30,31,32,33]. Some authors proposed a focused schlieren system by transforming a classical schlieren set-up to obtain quasi-planar measurements [5,23,29]. Other variants of the focused schlieren system were described in References [30,31], however, these systems had a limited field of view imposed by the optical components used on the experimentation. In another approach, a light source illuminating a source grid and a Fresnel lens were used to improve the method [32,33]. In a recent research, a structured light-field focusing technique was proposed to visualize fluid flows [34]. This approach stems for using light-field physics and multiple light sources to generate two-dimensional planar focused images. Also, this approach is simple to implement, flexible to applied to various fluid flows conditions and the process of alignment is easy to carry out. Indeed, most of the studies about focusing schlieren techniques have been used to visualize the behavior of fluid flows under different circumstances [35,36]. Few of these optical arrangements have been used to quantify a variable of a fluid flow [37]. In this way, in the present work, an approach in which the measurement of temperature fields on a narrow region in fluid flows is proposed.

The proposed technique is based in a relation between the intensity values of each pixel in a focused schlieren image to the corresponding cutoff grid position measured at the exit focal plane of the optical system. The focusing schlieren images are acquired using a focusing schlieren optical system setup based on an off-axis circular illumination. The kind of illumination used in this work allows generating an expanded synthetic aperture that improves the depth of focus of the optical system. This focusing effect neglect out-of-focus features, so that region of interest can be enhanced. Because of this setup technique, the temperature fields in different planes along the radial position of the nozzle of a commercial Hot Air Gun Soldering Station Welding are measured.

## 2. Theoretical Development

A ray of light suffers a deviation in its trajectory when passes through a not uniform medium. It is deviated a small angle [2,3,38]. The deviation angle depends on thickness and refractive index of the medium under study. The light ray deviation can be written as:

(1) $$\varepsilon_{\xi }=\int_{\zeta_{1}}^{\zeta_{2}}\frac{\partial n}{\partial \xi }dz,$$

where *n* = *n* (*x*, *y*, *z*) is the refractive index of the medium and *ξ* can be “*x*” or “*y*” axis. The axis direction is chosen according to the direction in which the cutoff grid blocks out the light. In this research a study is realized for the *x*-direction.

We substitute the Gladstone-Dale’s equation, (*n* − 1) = *Kρ*, in Equation (1) *and* we obtain the following expression:

(2) $$\rho_{x}=\frac{\partial \rho }{\partial x}=\frac{\delta x}{f\mathrm{hK}},$$

where *ρ* is the gas density, *h* is the thickness (region of interest in focus) of the inhomogeneous medium under test in the direction of ray propagation, *f* is the focal lens of the schlieren lens and *K* is the Gladstone-Dale’s constant. 

The Gladstone-Dale’s constant depend of the wavelength of the light source and the physical properties of the gas. In this work, considering our experimental conditions, we take a value of 2.271 × 10^−4^ m^3^/kg, for an air temperature of 293 K and a wavelength of 530 nm [2]. The temperature for an ideal gas at constant pressure can be found by using the following equation [39]:

(3) $$T=\frac{\rho_{o}}{\rho }T_{o}\frac{n_{o}-1}{n-1}T_{o}.$$

In Equation (3), *n_o_* and *ρ_o_* are the refractive index and density at reference temperature *T_o_*, respectively and *T* is the desired temperature. Therefore, our goal is obtaining the value *δx* of Equation (2) by using a calibration procedure [16,18]. In Section 4, a brief description of the method used to measure temperature fields by using a focusing schlieren optical system is introduced.

## 3. The Focusing Schlieren Method Base on an Off-Axis Circular Illumination

**Figure 1:**
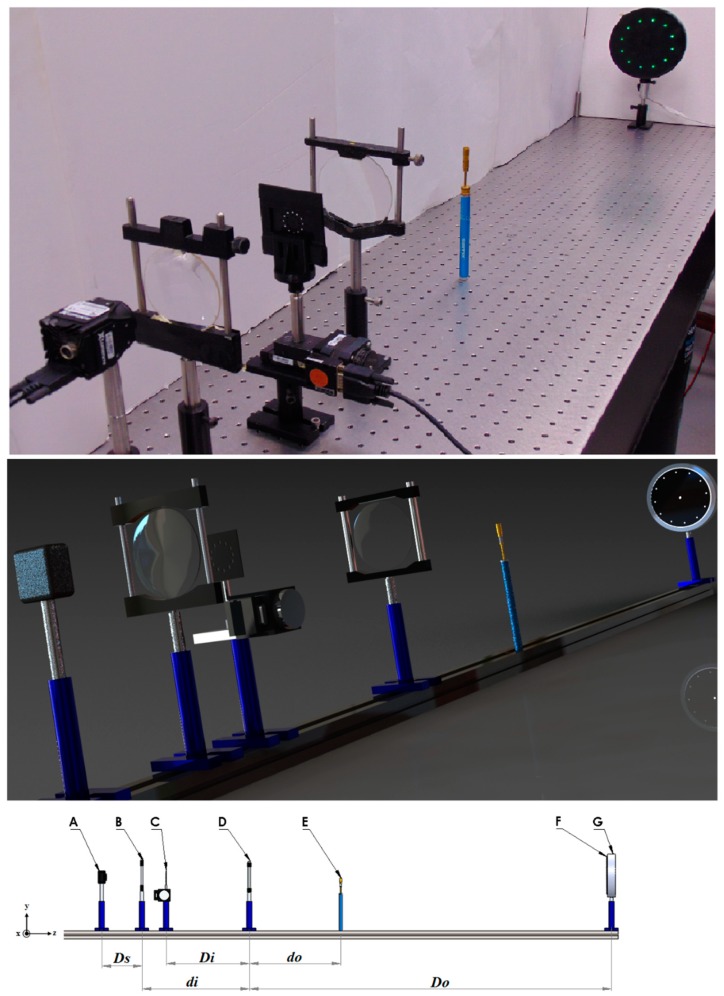
Scheme and picture of a focusing schlieren system based on an off-axis circular illumination.

Figure 1 shows the focusing schlieren system used in this work [34]. The optical system contains a source grid (F-G), a schlieren lens (D), a cutoff grid (C) and a digital camera (A-B). The sample test (E) is placed between the source grid and the schlieren lens. The source grid is a two-dimensional discrete light source array. It was configured using a green light circular lamp (G) of diameter 180 mm and a light aperture (F). The circular lamp has a power of 18 W and a luminous flux of 1200 lm. The light aperture is made of a 0.5 mm thick black plastic sheet and is stuck on the surface of the lamp. The light aperture contains 12, 3 mm diameter circular orifice positioned on a circular array. Each circular orifice is positioned off-axis from the center of the circular lamp at 60 mm. This array pattern configuration form an expanded synthetic aperture to reduce the Depth Of Focus (*DOF*) of the optical system. Also, there is a circular orifice of 3 mm diameter at the center of the light aperture used to test the *DOF* of the optical system illuminated with a source located on the optical axis.

In the system presented in this study, the schlieren lens (*f_#_* = 2.3) has a fixed focal length and a diameter of 215 mm and 90 mm respectively. The cutoff grid was made of the same material used to make the light aperture. The cutoff grid is scaled from the light aperture to a magnification of 0.641 given by the schlieren lenses. The orifices of the cutoff grid are square of side 1 mm and are arranged in a circular pattern. The cutoff grid was placed to emphasize density gradients in the stream-wise direction of the fluid flow. The object at the test area was imaged on the digital camera (A) by using a lens (B − *f_#_* = 1.7) with a fixed focal length of 128 mm and a diameter of 75 mm. When the optical system is aligned, the source grid at *D_o_* is an optical conjugate with the cutoff grid at *D_i_*. Also, the object plane at *d_o_* is an optical conjugate with the image position at *d_i_*. The positions of the conjugate planes can be determined by the thin lens equation [40].

The optical elements used in our set-up allow us to calculate the parameters needed to test the performance of the method. The system allows to have a Field Of View (*FOV*) of 17.4 × 9.2 mm^2^. The *DOF* of the focusing-schlieren system for one source [34] is written as follows

(4) $$DOF=\frac{2d_{o}f^{2}cf_{\#}\left(d_{o}-f\right)}{f^{4}-c^{2}f_{\#}^{2}{\left(d_{o}-f\right)}^{2}}$$

where *c* is the confusion circle, *f*_#_ = *f*/*d* and *f* and *d* are the focal distance and diameter of the schlieren lens respectively. The modified *f* - number for an off-axis source can be expressed as:

(5) $$f_{\#}=\frac{f}{d+\left(2\cdot OS\right)}$$

where *OS* is the off-axis distance from the axis of the optical system to a source of the source grid. The effective *DOF* of the system depends of the number of sources. Then, a lineal equation expressing this relation can be written as [33],

(6) $$DOF_{S}\approx \frac{DOF}{Ns}$$

where *Ns* is the number of sources. In our approach, the calculations of the *DOF* were done for three cases, that is, one source and six and twelve sources. The number of sources needed for the experiment was realized by maintaining uncovers one, six and twelve orifices of the source grid. The *DOF* for the one source case was estimated by maintaining uncover only the orifice located at the center of light aperture. 

**Figure 2:**
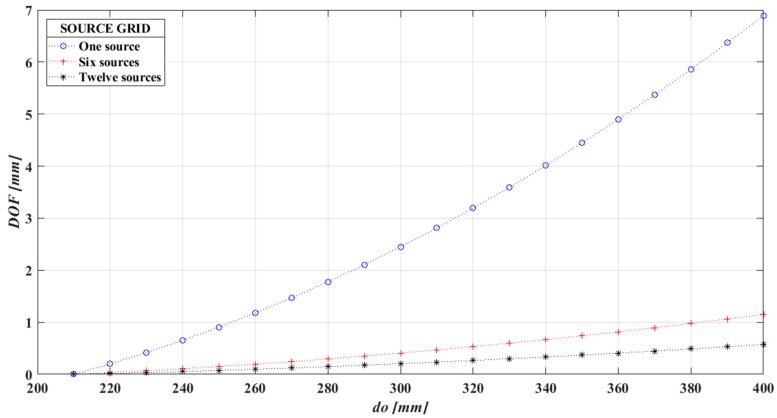
Theoretical performance of the focusing schlieren system used in this work.

From Equations (4)–(7), we can infer that high sensitivity and a narrow *DOF* imaging system requires a large *f* and small *f_#_* of the schlieren lens. Figure 2 shows the behavior of the *DOF* in function of the object plane position and the number of sources. Note that the *DOF* is smaller as the object plane approaches to the focus plane and because of the number of sources. For these calculations was used a circle of confusion of *c* = 2 mm [33]. It is important to notice that these values are theoretical, determined by ideal equations and some discrepancies can be obtained in the experimental results.

**Figure 3:**
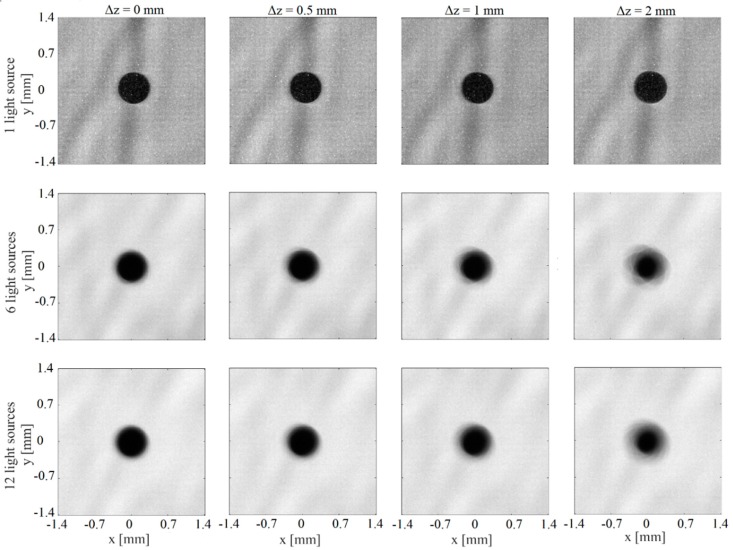
Focusing effect of a dot of a calibration slide. Top images: one source. Middle images: six sources. Bottom images: twelve sources. The images are registered moving the calibration slide away from the camera.

The performance of the system is tested experimentally by imaging a 0.6 mm dot of a calibration slide. The calibration dot is useful to accurate characterization of the focusing effect. Figure 3 shows the *DOF* effect of a dot of a calibration slide by using one, six and twelve light sources. The calibration slide was fixed to a mechanical device for a proper displacement. For a better comparison, a small section of the entire image was selected. In Figure 3, the top, middle and bottom images correspond to the *DOF* test using one, six and twelve light sources respectively. In our analysis “*z*” is measured along the optical axis and the slide is moving away from the camera. The focusing schlieren images were obtained at various calibration slide positions. We can observe that in the first case (one light source), the shape of the dot images at *z* = 0.0, 0.5 mm, 1.0 mm and 2.0 mm appear very well defined. Indeed, it is very difficult to find differences between the four images. The experimental *DOF* calculated for this case was of ~4.6 mm. In the two remaining cases, the shape of the dot was clearly visible in the plane of best focus (0 mm) and barely blurred at *z* = 0.5 mm. On the other hand, the shape of the dot was blurred at the planes *z* = 1.0 mm and 2.0 mm. In these two last cases, the value of the experimental *DOF* obtained was of ~0.8 mm and ~0.4 mm for six and twelve sources respectively. 

The experiments developed in this section tested the performance of the optical system used in this work by way of specific characteristics of the optical components. In this way, the system with twelve light sources offers a narrower *DOF* and it can be used to measure planar temperature fields of a commercial Hot Air Gun Soldering Station Welding.

## 4. Method to Measure Temperature in Fluid Flows 

The basic idea behind the procedure to measure temperature in fluid flows is to relate the intensity level of each pixel of a focused schlieren image to the corresponding cutoff grid position at the exit focal plane of the schlieren lens [16,18]. The transverse position of the cutoff grid should cover the conditions from maximum to minimum intensity of the light source. This methodology allows to trace a calibration curve which is subtracted to the focused schlieren image obtained when the cutoff grid is at the reference position (*I_o_*). The reference position represents the condition when the cutoff grid is at an intermediate value between the maximum and minimum light intensity, that is, when the intensity value is about 50% of the light observed for the condition of maximum intensity.

**Figure 4:**
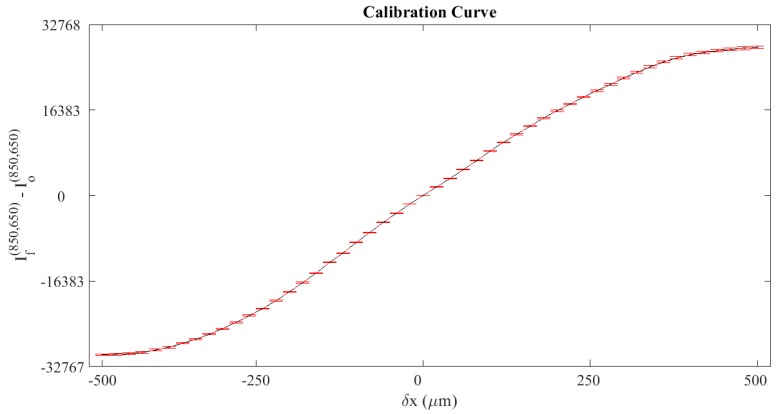
A typical calibration curve of a pixel and deviation along the curve.

The calibration curves obtained depict intensity deviations versus cutoff grid position (*δx*). Figure 4 represents a common calibration curve. In the same figure the deviation along the calibration curve is showed. An uncertainty value of ~80 intensity levels is obtained. This value is acceptable estimating the maximum intensity level of the calibration curves.

A calibration curve represents an operation equivalent to subtract a focused schlieren image in presence of flow (*I_f_*) from the focused schlieren image with no flow or at undisturbed conditions (*I_o_*). Thus, when a focused schlieren image in presence of flow (*I_f_*) is subtracted from the reference focused schlieren image (*I_o_*), the resulting value of each pixel is searched in the corresponding calibration curve to assign a quantity. In this way, the *ρ_x_* value can be determined via Equation (2) and the density value can be inferred by using the following Equation [3]:

(7) $$\rho (x)=\rho_{0}+\frac{1}{f_{}hK}\int_{\zeta_{1}}^{\zeta_{2}}\delta xdx.$$

The trapezoidal rule for integration is used to solve Equation (7). In references [16,18] is included a description of the procedure used in this work to determine temperature measurements.

## 5. Experiment

The goal in this experiment was to measure temperature fields of the hot air issuing from a 10 mm diameter nozzle of a commercial Hot Air Gun Soldering Station Welding (HAGSSW). The Hot air gun soldering station welding is a programmable digital display iron soldering. The programmable soldering allows induction of air temperatures from 100 °C up to 600 °C. In our experiments, the HAGSSW was set to temperature values of 200 °C and 400 °C. These values were corroborated by utilizing a K-type thermocouple probe provided by Fluke. The thermocouple was positioned near the exit of the nozzle of the soldering station. 

A focusing schlieren system was used in this research. The technical characteristics of the optical setup were introduced in Section 3. Figure 1 shows a schematic of the experimental arrange. The nozzle of the HAGSSW was positioned horizontally on an optical table. The stream-wise direction of the fluid flow was at *y*-direction and the cutoff grid was moved perpendicular to this direction. The temperature measurements of the hot air issuing from the nozzle were done in three planes, that is, *z* = 0, ±5 mm. 

The focusing schlieren images were acquired with a Lumenera color digital camera model Lt225. The camera is a 2.2 Megapixel CMOS sensor. It can provide 170 frames per second at full resolution and it has a pixel size of 5.5 μm. The captured images were saved in BMP format and digitized at 16-bit color intensity level. The camera is driven by its own software and allows the taking of either a single frame or a set of them. In the experiment just the images of the green color-channel were used. The spatial resolution of the camera allows registering of the main characteristics of the fluid flow under study.

The main aim of the experiment consists in recording focusing schlieren images with no flow for several transverse positions of the cutoff grid covering the conditions of maximum and minimum light intensity. The images captured were used for calibration purposes as described in Section 4. After that, a total of 100 focused schlieren images with flow for each case under study were registered. The measurements were taken at a room temperature of 23 °C.

## 6. Data Analysis

In order to show the viability of the method, the temperature fields from the experimental data outlined in Section 5 of the hot air issuing from the nozzle of the HAGSSW were determined.

### Temperature Data Analysis

The value of *ρ_x_* was determined by using the calibration procedure described in Section 4. Integration of this quantity is required to obtain the value of *ρ* as shown in Equation (7). The resulting density values were substituted in Equation (3) in order to obtain the temperature of interest.

**Figure 5:**
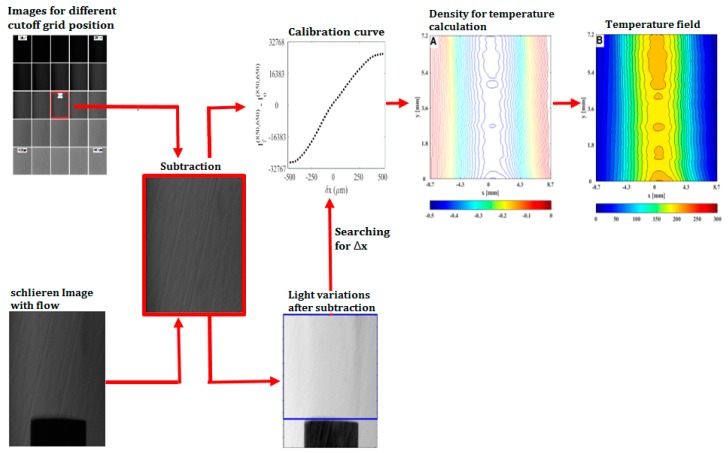
The flow chart for temperature measurements of fluid flows by using a focusing schlieren system.

**Figure 6:**
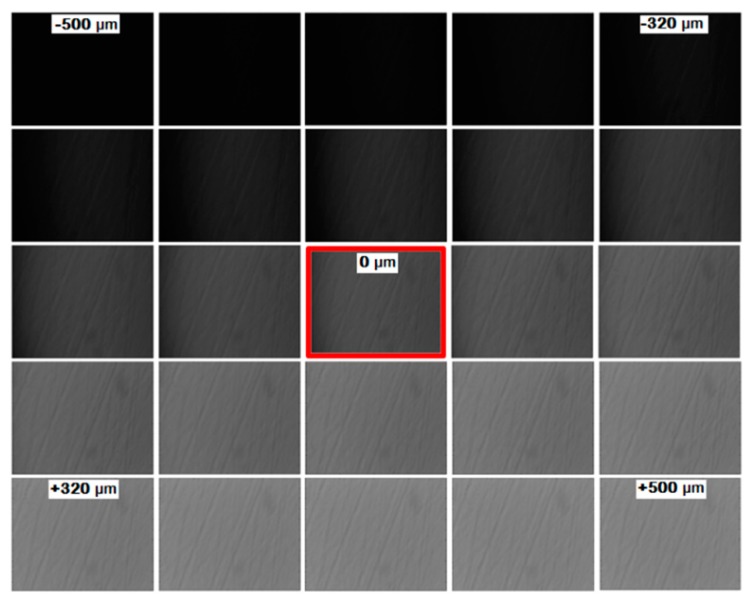
Focusing schlieren images for different cutoff grid positions for calibration purposes.

In Figure 5, a flow chart of the procedure is depicted. To obtain calibrations curves for each pixel, the cutoff grid was laterally displaced from −500 μm to 500 μm with a step size of 20 μm and at each cutoff grid position, a focused schlieren image was recorded. Figure 6 shows calibration images for different cutoff grid positions. All the focused schlieren images were subtracted from the image with the cutoff grid in its reference position (upper path in the flow chart). In the flow chart and Figure 6, the focused schlieren image with the cutoff grid in its reference position is indicated by a red square. On the lower path of the flow chart, a focused schlieren image in presence of flow, with the cutoff grid at its reference position, is shown. This image was subtracted from a focused schlieren image with no flow and the cutoff grid at its reference position. Therefore, the light deviation intensity for each pixel is directly related to its corresponding calibration curve. In this way, intensity deviation values of all pixels of each image are converted to cutoff grid displacement.

**Figure 7:**
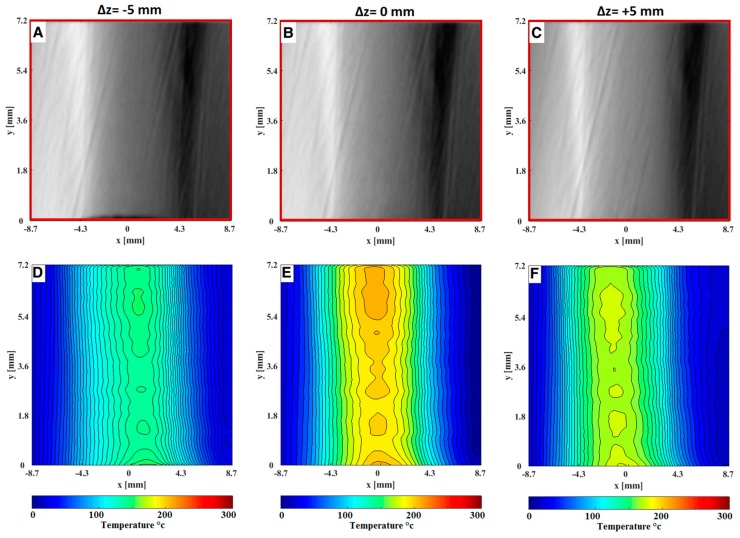
Instantaneous focusing schlieren images of the hot air issuing from the nozzle of the HAGSSW at 200 °C. A, B and C are the focusing schlieren images after convert each intensity level of pixels to cutoff grid displacements (*δx*) at planes *z* = −5 mm, *z* = 0 mm, *z* = 5 mm respectively. D, E and F are the corresponding temperature field at planes *z* = −5 mm, *z* = 0 mm, *z* = 5 mm respectively.

**Figure 8:**
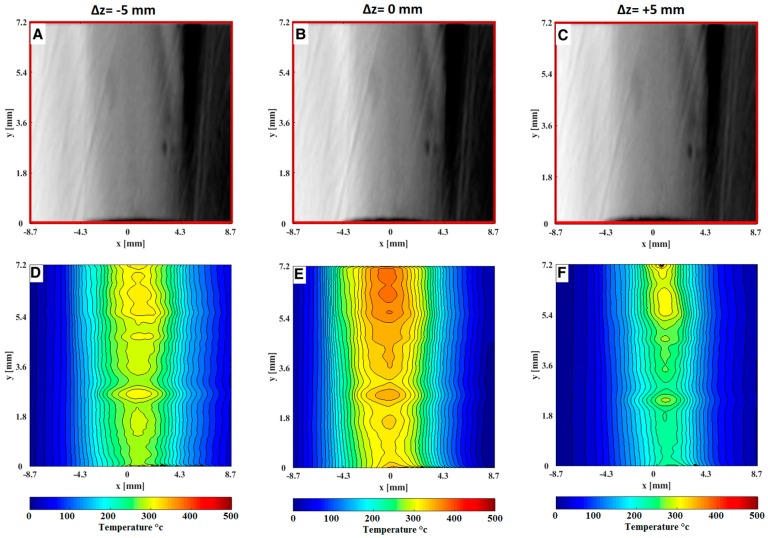
Instantaneous focusing schlieren images of the hot air issuing from the nozzle of the HAGSSW at 400 °C. A, B and C are the focusing schlieren images after convert each intensity level of pixels to cutoff grid displacements (*δx*) at planes *z* = −5 mm, *z* = 0 mm, *z* = 5 mm respectively. D, E and F are the corresponding temperature field at planes *z* = −5 mm, *z* = 0 mm, *z* = 5 mm respectively.

Figure 7 and Figure 8 show instantaneous focused schlieren images after converting to *δx* displacement and its corresponding temperature fields of the hot air issuing from the nozzle of the HAGSSW. In the images there are some imperfections caused by the schlieren lens, however, this *x* does not affect the calculation of the temperature. Also, notice the differences of the temperature fields at symmetrical planes, that is, *z* = ±5 mm. These discrepancies are due to non-uniformity in the fluid flow. Indeed, the optical system presented in this study is useful for characterizing the operation of the commercial HAGSSW and for non-symmetrical fluid flows.

**Figure 9:**
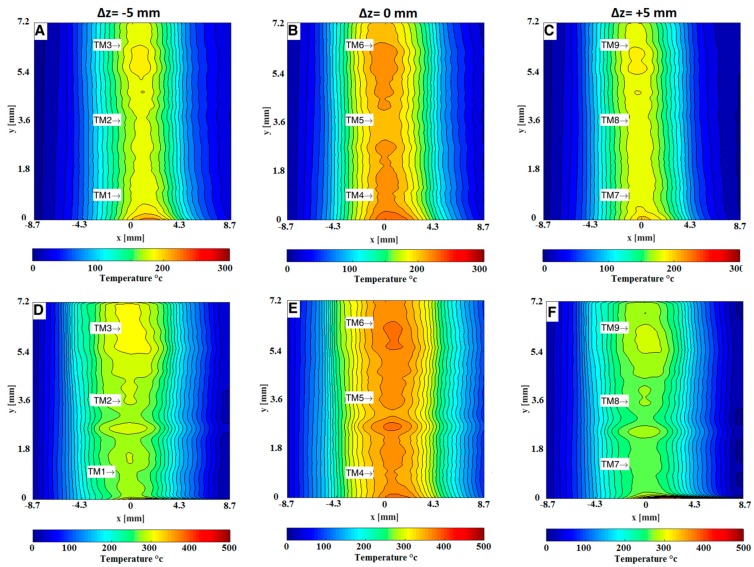
Average temperature fields utilizing 100 focusing schlieren images of the HAGSSW. A, B and C represent first case (200 °C) at planes *z* = −5 mm, *z* = 0 mm, *z* = 5 mm respectively. D, E and F represent second case (400 °C) at planes *z* = −5 mm, *z* = 0 mm, *z* = 5 mm respectively. TM: means, location and thermocouple measurement.

It was found that the temperature near the exit of the nozzle of the HAGSSW agreed with the temperature values selected at the programmable HAGSSW. Figure 9 shows average temperature fields for the two different temperature values under analysis. One hundred focused schlieren images were used to obtain the temperature average values. These temperature values were corroborated by a thermocouple measurement located at different points along the nozzle of the HAGSSW as is indicated on the Figure 9. Table 1 shows thermocouple and focusing schlieren temperature measurements at different points. The first and fifth columns represent the points indicated in Figure 9, the second and sixth columns are the thermocouple measurements for each case under analysis. The corresponding experimental measurements are displayed in columns three and seven. The fourth and eighth columns depict the relative error of each measurement point for each case under study. We notice that the relative error is very variable. However, the minimum and the maximum values are between 0.5% and 13.6%. These differences in measurements can be due to several factors such as fluid flow fluctuations, lens aberrations, radiation and convection effects present on the thermocouple measurement and errors inherent of the measurement process.

The approach presented in this work allows to obtain a minimum local temperature value of ~23 °C. On the other hand, the experimental data obtained in this study to calculate standard deviation for each case under study are used. The uncertainty was computed for measurements near and center of the exit of the nozzle of the soldering station, that is, at *z* = 0. We select this position since we expect to have lower fluid flow fluctuations. We obtained uncertainty values of 2.5 °C and 3.7 °C of the hot air issuing of the HAGSSW nozzle set at temperature values of 200 °C and 400 °C respectively.

The optical system presented in this study offer some specific characteristics that limit the range of temperatures to be measured and the kind of object to be analyzed. In order to change the capabilities of our optical system, it can be used optical components to have a wider FOV, high quality lenses to increase the accuracy of temperature measurements and high-speed digital cameras to study high speed phenomena. 

## 7. Conclusions

A planar temperature measurement of the hot air issuing from a nozzle of a Hot Air Gun Soldering Station Welding are reported. A simple focusing schlieren system based on an off-axis illumination was used for that purpose. The use of an off-axis illumination allows to focus in a narrow region of the fluid flow. This focusing effect neglect out-of-focus features, so that region of interest can be enhanced. Thus, the off-axis illumination used in this work permits to generate a larger synthetic aperture than the actual aperture of the schlieren lens. The approach allows to obtain a narrow depth of focus of the order of ~0.4 mm useful to attain planar temperature measurements in fluid flows. Also, in order to measure temperature fields, the proposed method makes use of a calibration procedure that allows to directly convert intensity level for each pixel of a focused schlieren image into a corresponding transverse cutoff grid position. The approach proposed in this work also is useful to explore a three-dimensional temperature field by incrementally visualizing individual planes along the optical path but perpendicular to it. The temperature values results are in agree with the thermocouple values. In future work, the system will be improved by using a high-speed digital camera, high quality lenses with of different optical parameters and different kind of illumination sources. The aim is to develop a digital focusing schlieren system that uses larger format lenses to reach greater sensitivity and larger fields of view.

## Figures and Tables

**Table 1:** Thermocouple and focusing schlieren temperature measurements.

Soldering Station at (200 °C)	Thermocouple (°C)	Focusing Schlieren (°C)	Relative Error (%)	Soldering Station at (400 °C)	Thermocouple (°C)	Focusing Schlieren (°C)	Relative Error (%)
TM1	178	176	1.0	TM1	297	312	5.0
TM2	172	166	3.5	TM2	330	346	4.8
TM3	174	171	1.7	TM3	328	333	1.5
TM4	201	219	9.0	TM4	330	356	5.7
TM5	212	215	1.4	TM5	384	392	2.0
TM6	213	221	3.7	TM6	372	370	0.5
TM7	170	178	4.7	TM7	238	261	9.6
TM8	175	184	5.1	TM8	249	283	13.6
TM9	181	186	2.7	TM9	274	290	5.8
